# GC-MS Studies on Nitric Oxide Autoxidation and *S*-Nitrosothiol Hydrolysis to Nitrite in pH-Neutral Aqueous Buffers: Definite Results Using ^15^N and ^18^O Isotopes

**DOI:** 10.3390/molecules28114281

**Published:** 2023-05-23

**Authors:** Dimitrios Tsikas

**Affiliations:** Core Unit Proteomics, Institute of Toxicology, Hannover Medical School, 30625 Hannover, Germany; tsikas.dimitros@mh-hannover.de

**Keywords:** autoxidation, derivatization, GC-MS, nitric oxide, pentafluorobenzyl bromide, stable isotopes

## Abstract

Nitrite (O=N-O^−^, NO_2_^−^) and nitrate (O=N(O)-O^−^, NO_3_^−^) are ubiquitous in nature. In aerated aqueous solutions, nitrite is considered the major autoxidation product of nitric oxide (^●^NO). ^●^NO is an environmental gas but is also endogenously produced from the amino acid L-arginine by the catalytic action of ^●^NO synthases. It is considered that the autoxidation of ^●^NO in aqueous solutions and in O_2_-containing gas phase proceeds via different neutral (e.g., O=N-O-N=O) and radical (e.g., ONOO^●^) intermediates. In aqueous buffers, endogenous *S*-nitrosothiols (thionitrites, RSNO) from thiols (RSH) such as L-cysteine (i.e., *S*-nitroso-L-cysteine, CysSNO) and cysteine-containing peptides such as glutathione (GSH) (i.e., *S*-nitrosoglutathione, GSNO) may be formed during the autoxidation of ^●^NO in the presence of thiols and dioxygen (e.g., GSH + O=N-O-N=O → GSNO + O=N-O^−^ + H^+^; p*K*_a_^HONO^, 3.24). The reaction products of thionitrites in aerated aqueous solutions may be different from those of ^●^NO. This work describes in vitro GC-MS studies on the reactions of unlabeled (^14^NO_2_^−^) and labeled nitrite (^15^NO_2_^−^) and RSNO (RS^15^NO, RS^15^N^18^O) performed in pH-neutral aqueous buffers of phosphate or t*ris*(hydroxyethylamine) prepared in unlabeled (H_2_^16^O) or labeled H_2_O (H_2_^18^O). Unlabeled and stable-isotope-labeled nitrite and nitrate species were measured by gas chromatography–mass spectrometry (GC-MS) after derivatization with pentafluorobenzyl bromide and negative-ion chemical ionization. The study provides strong indication for the formation of O=N-O-N=O as an intermediate of ^●^NO autoxidation in pH-neutral aqueous buffers. In high molar excess, HgCl_2_ accelerates and increases RSNO hydrolysis to nitrite, thereby incorporating ^18^O from H_2_^18^O into the SNO group. In aqueous buffers prepared in H_2_^18^O, synthetic peroxynitrite (ONOO^−^) decomposes to nitrite without ^18^O incorporation, indicating water-independent decomposition of peroxynitrite to nitrite. Use of RS^15^NO and H_2_^18^O in combination with GC-MS allows generation of definite results and elucidation of reaction mechanisms of oxidation of ^●^NO and hydrolysis of RSNO.

## 1. Introduction

Nitric oxide (^●^NO) is an environmental gas originating from many sources including combustion and thunderstorms. In living organisms, nitric oxide synthases (NOSs) are expressed virtually in all types of cell and convert L-arginine to L-citrulline and ^●^NO using molecular oxygen (^●^O^●^_2_) as the second substrate and many cofactors [1]. ^●^NO produced in cells such as endothelial cells needs to reach the soluble guanylyl cyclase in other cells such as the smooth muscle cells or platelets in order to exert biological effects. ^●^NO is a potent vasodilator and inhibitor of platelet aggregation and functions as a neurotransmitter [1]. ^●^NO may react with numerous intra- and extra-cellular biomolecules. Autoxidation of ^●^NO, i.e., its reaction with ^●^O^●^_2_, occurs immediately at the site of its generation, and this decreases the concentration of ^●^NO. NOS and many other enzymes generate reactive oxygen species (ROS) such as the superoxide anion (O_2_^●−^) and hydrogen peroxide (H_2_O_2_). O_2_^●−^ and H_2_O_2_ can react with ^●^NO before it can leave the cell. These reactions do not only decrease the concentration of ^●^NO, but they moreover produce reactive nitrogen species (RNS) such as peroxynitrite (ONOO^−^). Peroxynitrite is a strong oxidant and reacts with sulfhydryl (SH) groups in numerous low- and high-molecular-mass biomolecules.

Prior to the recognition of ^●^NO as an endogenous biomolecule, the oxidation of ^●^NO has been investigated in the gaseous (g) phase. The gas phase autoxidation of ^●^NO has the stoichiometry shown by Reaction (1) and Rate Law (2). Upon the recognition of ^●^NO as an endogenous signaling molecule about 35 years ago, the autoxidation of ^●^NO has been investigated in aqueous (aq) solutions [2,3,4,5,6,7,8,9]. The stoichiometry of the ^●^NO autoxidation in aqueous phases is given by Reaction (3) and its rate law by Expression (4) with 4*k*_aq_ = 9 × 10^6^ M^−2^s^−1^ at 25 °C [3]. Despite similar kinetics of the autoxidation of ^●^NO in the gas phase and in aqueous solutions, the reaction products differ: in aqueous solutions, nitrite is the sole autoxidation product of ^●^NO, whereas the reaction product formed in the gas phase is most likely ^●^NO_2_, which disproportionates upon dilution in aqueous solutions to nitrite and nitrate (5) [3]. In the presence of thiols, such as glutathione (GSH), in aqueous buffered solutions of ^●^NO and ^●^O^●^_2_, additional reaction products are formed. They include *S*-nitrosothiols or thionitrites (RSNOs) such as *S*-nitrosoglutathione (GSNO) and disulfides such as GSSG [10]. In the absence of ^●^O^●^_2_, neither GSNO nor GSSG formation has been observed. It has been hypothesized that not ^●^NO itself, but a ^●^NO-derived nitrosating intermediate, is formed, which reacts with GSH to form GSNO. This species has been proposed to be nitrous anhydride (N_2_O_3_) [10] (6), yet the structure of N_2_O_3_ has not been identified thus far [7], and the mechanisms of its formation in aqueous solutions are elusive. For N_2_O_3_, four isomeric structures have been suggested, including O=N-O-N=O and O=N-N^+^(=O)O^−^ [11]. Further proposed intermediates occurring during the autoxidation of ^●^NO include O=N-O-O^●^ and O=N-O-O-N=O [6,8,9]. Experiments performed at very low temperatures in non-aqueous systems, such as in glass-like matrixes of 2-methylbutane, suggested formation of yellow-colored (at 90 K, O=N-O-O^●^/^●^N=O and/or O=N-O-O^●^/O=N-O-O^●^) and red-colored (at 110 K, O=N-O-O-N=O or O=N-O-N(=O)O) intermediates [8,9]. Such species have not been detected in aqueous buffered solutions to date.

**Scheme 1:**
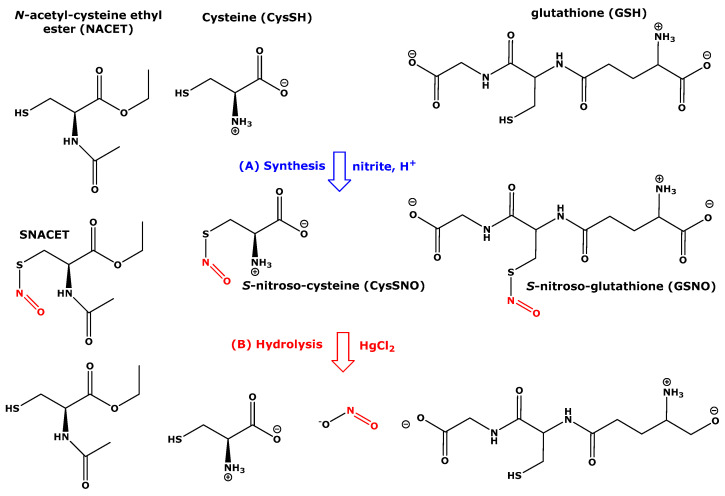
Simplified schematic presentation of (**A**) the chemical synthesis of *S*-nitroso-cysteinyl thiols (thionitrites, RSNO) from their thiols (RSH) and nitrite in aqueous solutions in the presence of diluted HCl acid and of (**B**) the HgCl_2_-induced hydrolysis of RSNO to the thiols and nitrite. CysSH, cysteine; GSH, glutathione; NACET, *N*-acetyl-cysteine ethyl ester. The corresponding *S*-nitrosothiols are *S*-nitroso-cysteine (CysSNO), *S*-nitroso-glutathione (GSNO), and *S*-nitroso-*N*-acetylcysteine ethyl ester (SNACET).

In the laboratory, RSNOs are prepared in aqueous solutions by mixing stoichiometric amounts of RSH and nitrite salts and by acidifying them with diluted acids such as HCl acid (Scheme 1) (7). Treatment of aqueous solutions of RSNO with a molar excess of an aqueous solution of HgCl_2_ leads to formation of nitrite (Scheme 1) (8). Both reactions are performed at room temperature. In cases of labile RSNO such as CysSNO, synthesis is preferably performed in an ice-bath (Scheme 1).

Generally, RSNOs are considered to be ^●^NO donors, yet this does not apply to every thionitrite. Thus, *S*-nitroso-L-cysteine (CysSNO) is an abundant “spontaneous” ^●^NO donor, whereas GSNO is not a ^●^NO donor. In phosphate buffer of neutral pH, as much as 50% of CysSNO may release ^●^NO [12], as can be specifically measured by NO-specific electrodes. The underlying mechanisms of ^●^NO release by RSNO are still incompletely resolved. Redox-active metal ions, most notably Cu^2+^/Cu^1+^, are extremely potent catalysts of the release of ^●^NO from CysSNO (9). Cu^2+^/Cu^1+^ are required in catalytic amounts and can be produced by small amounts of CysSH (10). It can, therefore, be assumed that the reaction products of *S*-nitrosothiols and possibly their intermediates in aqueous solutions may be different from those formed during the autoxidation of authentic ^●^NO.

2 ^●^N^(II)^=O**_(g)_** + ^●^O^(±0)●^_2_ → 2 ^●^N^(+IV)^O_2**(g)**_ (1)

−d[^●^NO]/dt = 2*k*_g_ × [^●^NO]^2^ × [O_2_] (2)

4 ^●^N^(II)^=O**_(aq)_** + ^●^O^(±0)●^_2_ + 2 H_2_O → 4 O=N^(III)^-O^−^ + 4 H^+^ (3)

−d[^●^NO]/dt = 4*k*_aq_ × [^●^NO]^2^ × [O_2_] (4)

2 ^●^N^(II)^O_2**(aq)**_ + H_2_O → O=N^(III)^-O^−^ + [O=N^(V)^(-O)-O]^−^ + 2 H^+^ (5)

GSH + N_2_O_3_ → GSNO + O=N-O^−^ + H^+^ (6)

RSH + O=N-O^−^ + H^+^ → RSNO + H_2_O (7)

2 RSNO + 2 H_2_O + HgCl_2(aq)_^+^ → Hg(RS)_2_ + 2 O=N-O^−^ + 2 Cl^−^ + 4 H^+^ (8)

CysSN^(III)^O + Cu^1+^ + H_2_O^+^ → ^●^N^(II)^O + CysSH + Cu^2+^ + OH (9)

CysS^(−II)^H + Cu^2+^ ←→ CysS^(−I)●^ + Cu^1+^ + H^+^ (10)

**Scheme 2:**
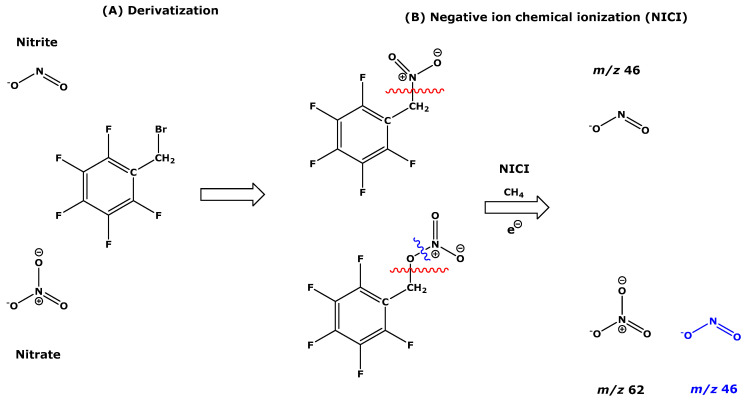
Simplified schematic of the (**A**) derivatization of nitrite and nitrate with pentafluorobenzyl (PFB) bromide to their nitro and nitric acid ester derivatives in aqueous solution, respectively, and (**B**) their negative-ion chemical ionization (NICI) in gas chromatography–mass spectrometry (GC-MS) to generate nitrite and nitrate, respectively. The PFB derivative of nitrate (PFB-ONO_2_) elutes before the PFB derivative of nitrite (PFB-NO_2_). Under NICI conditions, PFB-NO_2_ ionizes to form nitrite, whereas PFB-ONO_2_ ionizes to form nitrate (99.8%) and nitrite (0.2%) [13]. Methane is used as the reagent gas. *m/z*, mass-to-charge ratio.

In the present work, we investigated the reactions of L-cysteine-based RSNO and nitrite in aqueous buffers of neutral pH value by gas chromatography–mass spectrometry (GC-MS) in combination with the use of stable isotopes of O (natural abundance, 0.2% ^18^O) and N (natural abundance, 0.37% ^15^N) in RSNO, nitrite, and water (Scheme 1). The main analytes were unlabeled nitrite ([^14^N]nitrite), nitrite labeled with ^15^N ([^15^N]nitrite), and nitrite labeled with ^15^N and ^18^O (i.e., [^15^N, ^18^O]nitrite) (Scheme 2). The study provides strong indication for the formation of O=N-O-N=O as an intermediate during the autoxidation of ^●^NO derived from CysSNO in aqueous buffers of neutral pH value. In H_2_^18^O, ^●^NO autoxidizes to ^18^O-nitrite and ^16^O-nitrite. In aqueous solutions, HgCl_2_ mediates the hydrolysis of the SNO groups of CysSNO and GSNO to ^18^O-nitrite and ^16^O-nitrite.

## 2. Materials and Methods

### 2.1. Chemicals and Materials

Na^15^NO_2_ (98.5 atom% ^15^N) was from Cambridge Isotope Laboratories (Andover, MA, USA). Na^15^NO_3_ (98.5 atom% ^15^N) was from Sigma (Munich, Germany). ^18^O-Labeled water (95.5 atom% at ^18^O) was purchased from Campro-Scientific (Berlin, Germany). Tetramethylammonium peroxynitrite, [Me_4_N]^+^[ONOO]^–^, supplied as 1 mL portions of a 13.5 mM solution in 10 mM KOH (based on ε = 1700 M^−1^cm^−1^ at 302 nm in 10 mM KOH), was from Alexis (Grünberg, Germany). The stock solution of Me_4_N]^+^[ONOO]^–^ was stored at −80 °C. All peroxynitrite-containing solutions were kept on ice in the dark (aluminum foil). Peroxynitrite solutions were used immediately after thawing [Me_4_N]^+^[ONOO]^–^ without renewed refrigerating of the remaining sample. CysSH, GSH, GSSG, HgCl_2_, and pentafluorobenzyl (PFB) bromide were from Sigma-Aldrich (Munich, Germany). *N*-Acetylcysteine ethyl ester (NACET) was prepared as reported elsewhere [14]. K_2_HPO_4_, *tris*(hydroxymethyl)amino methane (Tris) and concentrated hydrochloric acid were obtained from Merck (Darmstadt, Germany). These salts were used to prepare 100 mM and 200 mM buffers of pH 7.4, respectively. Stock solutions of *S*-nitrosothiols were freshly prepared by combining equal volumes of ice-cold 10 mM solutions of nitrite and the thiols in distilled water and acidifying the samples by adding 10 µL aliquots of ice-cold 5 M HCl solutions followed by brief vortex mixing [15]. These samples were stored in an ice-bath in aluminum foil to avoid light-induced decomposition of the *S*-nitrosothiols and were used on the same day to prepare dilutions in the buffers. Li^18^OH was prepared by adding a weighed amount of elemental Li (stored in paraffin) to a small volume of H_2_^18^O.

### 2.2. Experimental Conditions

All experiments were performed either in 100 mM K_2_HPO_4_ buffer or in 200 mM Tris buffer, both of pH 7.4, at room temperature (about 20–23 °C). For the sake of simplicity and comprehensibility, the experiments are described in detail in the Section 3.

### 2.3. Derivatization Procedure for Nitrite and Nitrate

Unlabeled and labeled nitrite and nitrate species were derivatized simultaneously with PFB bromide as described elsewhere [13] (Scheme 2), except for the sample volumes which varied (see Section 3). A constant sample–acetone volume ratio of 1:4 and a constant volume of toluene (1 mL) were used for the extraction of the PFB derivatives of the nitrite (PFB-NO_2_) and nitrate (PFB-ONO_2_) species.

### 2.4. GC-MS Analyses

Derivatized unlabeled and labeled nitrite and nitrate species were measured by GC-MS on an Agilent system model 5980 based on the quadrupole technology. An Optima 17 (15 m × 0.25 mm i.d., 0.25 µm film thickness) from Macherey-Nagel was used. Helium (70 kPa) and methane (200 Pa) were used as carrier and reactand gas, respectively. Aliquots (1 µL) of toluene extracts were injected in the splitless mode. Oven temperature was held at 70 °C for 1 min and then increased to 280 °C at a rate of 30 °C/min. Constant temperatures were kept at the ion source (180 °C), interface (280 °C), and injector (200 °C). Negative-ion chemical ionization (NICI) was used at an electron energy of 230 eV and an emission current of 300 µA (Scheme 2). Nitrite and nitrate species were analyzed in the selected-ion monitoring (SIM) mode using a dwell time of 50 ms for each ion (Table 1). The sum of peak area values of all ions monitored was set to 100%. Peak area values of selected ions were used to calculate their peak area ratio (PAR).

## 3. Results

### 3.1. Hydrolysis of CysSNO and GSNO in H_2_^18^O

A 200 µL aliquot of 200 mM Tris buffer, pH 7.4, was extensively evaporated to dryness under a stream of nitrogen gas. The solid residue was reconstituted in 200 µL H_2_^18^O. After vortexing (highest stage), the sample was divided into four 50 µL aliquots. Two samples were spiked with CysSNO to reach a final added concentration of 100 µM (sample A, sample B). Yet another two samples were spiked with GSNO to reach final added concentrations of 100 µM (sample C, sample D). Subsequently, 2 µL aliquots of a 10 mM solution of HgCl_2_ in deionized water (H_2_^16^O) were added to sample A and sample C at an approximate final concentration of 1 mM each. To allow complete HgCl_2_-induced decomposition of CysSNO and GSNO, the samples were incubated for 60 min at room temperature [15]. Samples B and D were incubated at room temperature for 3 h and 24 h, respectively, to allow for spontaneous decomposition of CysSNO and GSNO. At the end of the incubation, all samples were treated with PFB bromide to convert nitrite species to their PFB nitro derivatives. GC-MS analysis was performed by SIM of *m/z* 46, *m/z* 48, and *m/z* 50. The results of this experiment are summarized in Table 2.

In Tris buffer, the half-life for CysSNO is about 7 min [15]. Immediate treatment of the 100 µM solution of CysSNO (0 h) in ^18^O-Tris buffer with HgCl_2_ (sample A) resulted in the formation of ^18^O=N-O^−^/O=N-^18^O^−^ (*m/z* 48) and ^16^O=N-O^−^/O=N-^16^O^−^ (*m/z* 46) with a peak area ratio (PAR) of 1:1.4. In this experiment, the formation of ^18^O=N-^18^O^/18^O=N-^18^O^−^ (*m/z* 50) amounted to only 1%. This observation suggests that ^18^O from ^18^O-Tris buffer is incorporated into the SNO group of CysSNO induced by HgCl_2_.

In the case of sample B, i.e., in the absence of HgCl_2_, incubation resulted in the formation of ^18^O=N-O^−^/O=N-^18^O^−^ (*m/z* 48) and ^16^O=N-^16^O (*m/z* 46) with a PAR of 1:1.4. The formation of ^18^O=N-^18^O^−^ (*m/z* 50) amounted to 22%. This difference is likely to be due to the longer incubation time of 3 h and the absence of HgCl_2._ The incubation time of 3 h allows for complete decomposition of CysSNO to NO. The formation of *m/z* 46, *m/z* 48, and *m/z* 50 is likely to result in part by hydrolysis of the SNO group of intact CysSNO and in part due to autoxidation of CysSNO-derived to NO.

Incubation of GSNO in ^18^O-Tris buffer in the absence of HgCl_2_ (sample D) did not result in formation of ^18^O=N-O^−^/O=N-^18^O^−^ (*m/z* 48) to an appreciable extent. This observation suggests that GSNO does not release NO nor hydrolyzes to ^18^O=N-O^−^/O=N-^18^O^−^ in ^18^O-Tris buffer. In contrast, GSNO immediately treated with HgCl_2_ (sample C) resulted in the formation of ^18^O=N-O^−^/O=N-^18^O^−^ (*m/z* 48) to an even greater extent compared to unlabeled nitrite (67% vs. 30%). Obviously, HgCl_2_ is required for the hydrolysis of the SNO group of GSNO to nitrite.

### 3.2. Hydrolysis of CysS^15^N^18^O and GS^15^N^18^O in H_2_^16^O

The experiment described above was repeated with some modifications. Separate solutions (100 µL) of [^15^N]nitrite, CysSH, and GSH were prepared in 200 mM Tris buffer, pH 7.4. Then, the solvent was evaporated thoroughly under a stream of nitrogen. Subsequently, the [^15^N]nitrite samples were reconstituted in 100 µL aliquots of H_2_^18^O, and these solutions were used to reconstitute the CysSH and GSH residues. CysS^15^N^18^O and GS^15^N^18^O were synthesized separately in these samples by adding 2.5 µL aliquots of 5 M HCl. After incubation for 5 min to complete CysS^15^N^18^O and GS^15^N^18^O, the samples were neutralized (pH 7 to 8) by adding 2.5 µL aliquots of 5 M Li^18^OH. Then, the CysS^15^N^18^O and GS^15^N^18^O samples were each divided into two 50 µL aliquots. Immediately thereafter, one CysS^15^N^18^O sample (sample A) and one GS^15^N^18^O sample (sample C) were each spiked with 10 µL of a 10 mM solution of HgCl_2_ prepared in H_2_^18^O. The second CysS^15^N^18^O sample (sample B) and the second GS^15^N^18^O sample (sample D) were incubated at room temperature for 3 h and 24 h, respectively, to allow complete decomposition of these RSNOs. In addition, two [^15^N]nitrite samples were used as controls. One [^15^N]nitrite sample (sample E) was incubated for 5 min at room temperature in 200 mM ^18^O-Tris buffer, pH 7.4. The other [^15^N]nitrite sample (sample F) was incubated at room temperature in acidified (about pH 2) 200 mM ^18^O-Tris buffer. After PFB bromide derivatization, GC-MS analysis was performed for nitrite species by SIM. The results of this experiment are summarized in Table 3.

Immediate treatment of CysS^15^N^18^O (sample A) and GS^15^N^18^O (sample C) with HgCl_2_ (H_2_^18^O) resulted in almost complete hydrolysis of the RSNO and formation of ^18^O=^15^N-^16^O^/16^O=^15^N-^18^O^−^ (*m/z* 49) and of ^18^O=^15^N-^18^O^−^ (*m/z* 51) with a PAR *m/z* 51 to *m/z* 49 of 1:1 for both RSNOs. Comparable results were obtained from the incubation of CysS^15^N^18^O in the absence of HgCl_2_ (sample B). In the case of sample D, GS^15^N^18^O (24 h) resulted in the formation of ^18^O=^15^N-^16^O^−/16^O=^15^N-^18^O^−^ (*m/z* 49) and of ^18^O=^15^N-^18^O^−^ (*m/z* 51) with a PAR of 2.3:1, suggesting higher incorporation of ^18^O into the S^15^N^18^O group of GS^15^N^18^O compared to CysS^15^N^18^O.

For comparison, [^15^N]nitrite was incubated in non-acidified ^18^O-Tris buffer (pH 7.4, sample E) and in acidified ^18^O-Tris buffer (pH 2.0, sample F). In non-acidified ^18^O-Tris buffer, there was little incorporation of ^18^O into [^15^N]nitrite, whereas the incorporation of ^18^O into [^15^N]nitrite in acidified ^18^O-Tris buffer (pH 2, sample F) was almost complete. Thus, in ^18^O-Tris buffer (pH 7.4) a small incorporation of ^18^O from Tris buffer into [^15^N]nitrite is possible, yet it is lower than in RSNO. Nitrite is the conjugate base of nitrous acid (HONO; p*K*_a_, 3.2), and HONO and/or its anhydride seems to be more easily accessible for hydrolysis than nitrite and RSNO.

### 3.3. HgCl_2_-Induced Hydrolysis of NACCysS^14^NO and NACCysS^15^NO in H_2_^16^O/H_2_^18^O Mixtures

In a further experiment, the HgCl_2_-induced hydrolysis of NACCysS^14^NO and NACCysS^15^NO was investigated in phosphate buffer of pH 7.4 using HgCl_2_ prepared in H_2_^16^O/H_2_^18^O mixtures.

An equimolar mixture of NACCysS^14^NO and NACCysS^15^NO was diluted in 100 mM K_2_HPO_4_ buffer, pH 7.4, to reach a final concentration of 238 µM. Aliquots (12.5 µL) of this solution were treated with 12.5 µL aliquots of HgCl_2_ solutions prepared in H_2_^16^O/H_2_^18^O mixtures. The H_2_^16^O/H_2_^18^O mixtures varied (*v/v*) as follows: sample A: 100:0; sample B, 100:5; sample C, 100:25; sample D, 100:100; sample E, 100:75; and sample F, 0:100. The final concentration of HgCl_2_ was constant at 3.33 mM. All samples were incubated for 10 min at room temperature and then derivatized with PFB bromide. GC-MS analysis was performed in the SIM mode. The results of this experiment are summarized in Table 4.

The ^15^N- to ^14^N-nitrite molar ratio (*m/z* 47 to *m/z* 46) was independent of the H_2_^16^O/H_2_^18^O final volume ratio in the samples and was determined to be 1.070 ± 0.045 (mean ± SD, *n* = 5). The molar ratios of *m/z* 48 to *m/z* 46 and of *m/z* 49 to *m/z* 47 increased with an increasing proportion of H_2_^18^O in the samples. The increase was linear until a proportion of 37.5% of H_2_^18^O in the sample. These observations suggest that ^18^O from H_2_^18^O is incorporated almost to the same extent into ^15^N- to ^14^N-nitrite released from NACCysS^15^NO and NACCysS^14^NO, respectively.

In a further experiment, separate solutions of ^14^N-nitrite (500 µM), GS^14^NO (238 µM), and NACCysS^14^NO (100 µM) were prepared in 100 mM K_2_HPO_4_ buffer, pH 7.4, using H_2_^16^O. From these solutions, 12.5 µL aliquots were treated with 12.5 µL aliquots of a 5 mM HgCl_2_ solution prepared in H_2_^18^O. The final H_2_^16^O:H_2_^18^O volume ratio was constant at 1:1 (*v/v*). Derivatization and GC-MS analyses of these samples resulted in a PAR *m/z* 46 to *m/z* 48 of 51:1 for ^14^N-nitrite, 1.25:1 for GS^14^NO, and 1.84:1 for NACCysS^14^NO. These data suggest considerable incorporation of ^18^O from H_2_^18^O into ^14^N-nitrite derived from GS^14^NO and NACCysS^14^NO, but not into authentic ^14^N-nitrite.

### 3.4. Decomposition and Isomerization of Synthetic Peroxynitrite in H_2_^18^O

Similar experiments were performed with freshly prepared dilutions of commercially available peroxynitrite (i.e., tetramethylammonium peroxynitrite; 100 µM) in 0.2 M Tris buffer and in 0.1 M potassium phosphate buffer (each of pH 7.4). They resulted in formation of ^18^O-labeled nitrite and ^18^O-labeled nitrate to the same very low extent, closely comparable to that obtained using solutions of synthetic nitrite and nitrate (each at 100 µM) in pH-neutral 0.2 M Tris buffer and 0.1 M potassium phosphate buffer. ^18^O incorporation was very low even in the buffers prepared in 100% H_2_^18^O for long incubation times (up to 60 min). These results suggest that water is not involved in the decomposition of peroxynitrite to nitrite and isomerization of peroxynitrite to nitrate in aqueous buffers of neutral pH value. Synthetic peroxynitrite was found to decompose to nitrite and to isomerize to nitrate with a stoichiometry of 1:1 [16] (11). Decomposition of peroxynitrite to nitrite and dioxygen with a stoichiometry of 2:1 been reported by others [17].

4 [O=N^(III)^-O^(−I)^-O^(−I)^]^−^ → 2 [O=N^(III)^-O^(−II)^]^−^ + 2 [O=N^(V)^(-O^(−II)^)-O^(−II)^]^−^ + O^(±0)^_2_ (11)

### 3.5. Effects of GSH on Reaction of Synthetic Peroxynitrite in H_2_^18^O

GSH and other thiols such as CysSH react with peroxynitrite [15]. Known reaction products of peroxynitrite and GSH are GSNO, oxidized GSH, i.e., GSH disulfide (GSSG), nitrite, and nitrate. As shown above, in the absence of GSH, peroxynitrite decomposes to nitrite and isomerizes to nitrate. In the presence of GSH, peroxynitrite increasingly decomposes to nitrite at the cost of its isomerization product nitrate (11).

The reaction of peroxynitrite with GSH was investigated in aqueous buffers prepared in H_2_^18^O. At a concentration of 5 mM GSH, no incorporation of ^18^O from H_2_^18^O into nitrite or nitrate from decomposed peroxynitrite (100 µM) was observed. For comparison, the same experiment was performed with nitrite (100 µM) instead of peroxynitrite. Linear regression analysis between the PAR of *m/z* 48 to *m/z* 46 *(y*_1_) or the PAR of *m/z* 50 to *m/z* 46 (*y*_2_) and the percentage content of H_2_^18^O (x) was performed. The regression equations were *y*_1_ = 2 × 10^−3^ + 4.9 × 10^−4^ x (*r*^2^ = 0.984) and *y*_2_ =2 × 10^−5^ + 1.9 × 10^−4^ x (*r*^2^ = 0.944) for peroxynitrite. The corresponding regression equations were *y*_1_ = 4 × 10^−3^ + 5.3 × 10^−4^ x (*r*^2^ = 0.9967) and *y*_2_ = 8 × 10^−5^ + 2.6 × 10^−5^ x (*r*^2^ = 0.958) for nitrite. These very similar results suggest that in aqueous buffer of neutral pH, peroxynitrite is converted to nitrite (decomposition) without the participation of water.

In the presence of GSH, the peroxy group of peroxynitrite is reduced to yield nitrite and GSSG via intermediate formation of GSOH (12a). GSOH further reacts with GSH to form GSSG (12b). GSSG is the major reaction product of GSH with peroxynitrite [16]. ^18^O=^14^N-^18^O^−^ (*m/z* 50) was formed from the reaction of GSH with peroxynitrite in H_2_^18^O to a very low extent which was, however, higher than the incorporation of ^18^O into authentic nitrite. This could be due to the occurrence of additional much less abundant reactions such as the formation of GSNO (12) and ^●^NO.

2 GS^(−II)^H + [O=N^(III)^-O^(−I)^O^(−I)^]^−^ → GS^(−I)^S^(−I)^G + [O=N^(III)^-O^(−II)^ ]^−^ + H_2_O (12)

GS^(−II)^H + [O=N^(III)^-O^(−I)^O^(−I)^]^−^ → GS^(±0)^OH + [O=N^(III)^-O^(−II)^]^−^ (12a)

GS^(−II)^H + GS^(±0)^OH → GS^(−I)^S^(−I)^G + H_2_O (12b)

## 4. Discussion

The elements H, C, N, and O are mixtures of stable isotopes. The natural abundance of their heavier isotopes amounts to 0.0145% ^2^H, 1.06% ^13^C, 0.366% ^15^N, and 0.2% ^18^O. Analytes “labeled” with stable isotopes of these elements can be separated from their “unlabeled” analogs by mass spectrometry (MS). Stable-isotope-labeled compounds are useful as internal standards in MS-based quantitative chemical analysis, because they have almost identical physicochemical properties. The only difference is the formation of ions with different mass-to-charge (*m/z*) ratios, which is utilized in mass spectrometers for their separation.

Another important topic of application of stable isotopes is qualitative and quantitative physical, chemical, biochemical, and biomedical research. The present work demonstrates the unique utility of the use of stable isotopes to perform mechanistic studies on reactions of ^●^NO and its metabolites *S*-nitrosothiols (RSNOs), peroxynitrite, nitrite, and nitrate and to obtain definite results. In these studies, H_2_^18^O was used in combination with a highly specific GC-MS method [13] for the simultaneous measurement of nitrite and nitrate species that contain ^14^N, ^15^N, ^16^O, or ^18^O in their molecules. The GC-MS method uses simultaneous derivatization of nitrite and nitrate in aqueous buffers with PFB bromide, methane negative-ion chemical ionization of the PFB derivatives to nitrite and nitrate, their separation on a single quadrupole GC-MS apparatus, and detection by an electron multiplier (Scheme 2). Nitrite and nitrate are ubiquitous, i.e., they are present as contaminations in the laboratory, at concentrations lying in the lower µM range. The high specificity and sensitivity of the GC-MS method and the relatively low natural abundance of ^15^N and ^18^O enable performance of experiments using small amounts (volumes) of H_2_^18^O and relatively small quasi-physiological concentrations of reactands. These features help overcome the ubiquity of nitrite and nitrate contaminations. H_2_^18^O is a quite expensive solvent. This is an issue and may limit the number of replicates. However, the information gained by such experiments overwhelms potential limitations.

**Scheme 3:**
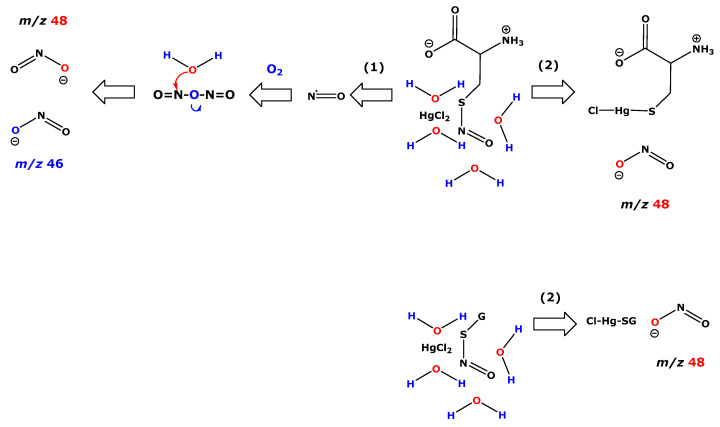
Proposed mechanisms for the incorporation of ^18^O from H_2_^18^O into (1) nitrous anhydride (N_2_O_3_) from autoxidized NO formed from decomposed CysSNO and into (2) the SNO groups of CysSNO (upper panel) and GSNO (lower panel) mediated by aqueous HgCl_2_. GSNO is not a NO donor. In its H_2_^18^O solutions, HgCl_2_ forms a hydratation shell with H_2_^18^O, which attacks the SNO groups of CysSNO and GSNO to form ^18^O-labeled nitrite.

The results presented in the current study unequivocally demonstrate that H_2_^18^O is involved in the generation of nitrite from RSNO, in part via autoxidation of ^●^NO and hydrolysis of the unisolable intermediate N_2_O_3_, the anhydride of nitrous acid. This is the case in CycSNO (Scheme 3). GSNO is neither a ^●^NO donor nor hydrolyzes to nitrite. In high molar excess, HgCl_2_ in aqueous solution mediates the hydrolysis of the SNO group of CysSNO and GSNO (Scheme 3) as well as of NACCysSNO and most likely of every RSNO. There is indication that the Hg^(II)^ ion in HgCl_2_ used in the experiments forms a hydratation shell with several H_2_^18^O molecules (Scheme 3). Literature reports support this observation, indicating that the first solvation shell of Hg^2+^ ions may contain 6 to 24 water molecules [18,19,20,21,22,23,24]. Experiments with H_2_^18^O should consider possible effects of hydratation of reagents used to prepare buffers and solutions of chemicals, which may potentially form stable hydratation shells with H_2_^16^O that are difficult to be completely displaced by H_2_^18^O.

## 5. Conclusions

Currently, simultaneous analysis of nitrite and nitrate is best performed by GC-MS after simultaneous derivatization with pentafluorobenzyl bromide and negative-ion chemical ionization. This is a unique technique to detect nitrite and nitrate anions as they occur in biological samples. The combination of this GC-MS approach with the use of buffers prepared in H_2_^18^O enables generation of definite results in mechanistic studies allowing elucidation. In H_2_^18^O buffers of neutral pH, *S*-nitroso-cysteine (CysSNO) decomposes to form ^18^O-nitrite, indicating the involvement of water. This is not the case for *S*-nitroso-glutathione (GSNO). HgCl_2_ mediates the hydrolysis of the SNO groups of CysSNO and GSNO. Aqueous solutions of HgCl_2_ are likely to form Hg^2+^ ions solvated with H_2_^16^O and H_2_^18^O, and this “isotope effect” may influence the outcome of hydrolysis studies.

## Figures and Tables

**Table 1:** Pentafluorobenzyl derivatives of nitrite and nitrate species quantitated by selected-ion monitoring of specific mass-to-charge (*m/z*) ions. The ^15^N isotope (natural abundance, 0.37%); the ^18^O isotope (natural abundance, 0.2%).

Species	*m/z*	Structure of the Anion
Nitrite	46	O=N-O^−^
	47	O=^15^N-O^−^
	48	^18^O=N-O^−^ or O=N-^18^O^−^
	49	^18^O=^15^N-O^−^ or O=^15^N-^18^O^−^
	50	^18^O=N-^18^O^−^ or ^18^O=N-^18^O^−^
	51	^18^O=^15^N-^18^O^−^ or ^18^O=^15^N-^18^O^−^
Nitrate	62	O=N(-O)-O^−^
	63	O=^15^N(-O)-O^−^
	64	^18^O=N(-O)-O^−^ or O=N(-O)-^18^O^−^
	65	^18^O=^15^N(-O)-O^−^ or O=^15^N(-O)-^18^O^−^
	66	^18^O=N(-O)-^18^O^−^ or ^18^O=N(-O)-^18^O^−^

**Table 2:** Incorporation of ^18^O from H_2_^18^O into nitrite upon incubation of unlabeled CysSNO and GSNO in ^18^O-prepared 200 mM Tris buffer, pH 7.4. Numbers in parentheses indicate the incubation time; the incubation time with HgCl_2_ was 1 h.

Sample	*m/z* 46 (%)	*m/z* 48 (%)	*m/z* 50 (%)	*m/z* 48/*m/z* 46
A CysSNO (0 h) + HgCl_2_	58	41	1	0.7:1
C GSNO (0 h) + HgCl_2_	30	67	3	2.2:1
B CysSNO (3 h)	45	33	22	0.7:1
D GSNO (24 h)	95	5	0	0.05:1

**Table 3:** Incorporation of ^18^O from H_2_^18^O into ^15^N-nitrite formed from CysS^15^N^18^O and GS^15^N^18^O in 200 mM Tris buffer, pH 7.4, prepared in H_2_^18^O.

Sample	*m/z* 47 (%)	*m/z* 49 (%)	*m/z* 51 (%)	*m/z* 51/*m/z* 49
A CysS^15^N^18^O (0 h) + HgCl_2_ (H_2_^18^O)	4	48	48	1:1
C GS^15^N^18^O (0 h) + HgCl_2_ (H_2_^18^O)	4	48	48	1:1
B CysS^15^N^18^O (3 h)	8	44	48	1.2:1
D GS^15^N^18^O (24 h)	2	30	68	2.3:1
E [^15^N]Nitrite (5 min), pH 7.4	78	19	3	0.16:1
F [^15^N]Nitrite (5 min), pH 2.0	8	37	56	1.5:1

**Table 4:** Incorporation of ^18^O from H_2_^18^O into ^14^N- and ^15^N-nitrite formed from NACCysS^14^NO and NACCyS^15^NO in 100 mM potassium phosphate buffer, pH 7.4, in the presence of a 3.33 mM HgCl_2_ solution in H_2_^16^O/H_2_^18^O mixtures. n.d., not detected.

Sample	H_2_^18^O (vol%)	*m/z* 47/*m/z* 46	*m/z* 48/*m/z* 46	*m/z* 49/*m/z* 47
		O^15^NO^−^/O^14^NO^−^	^18^O^14^NO^−^/O^14^NO^−^	^18^O^15^NO^−^/O^15^NO^−^
A	0	1.04	0.005	0.005
B	2.5	n.d.	0.064	0.065
C	12.5	1.03	n.d.	n.d.
D	25.0	1.05	0.432	0.435
E	37.5	1.09	0.642	0.688
F	50.0	1.14	0.676	0.744

## Data Availability

Not applicable.

## Sample Availability

Not available.
